# In vivo percutaneous microwave ablation with ECO system in swine kidney and liver: comparison of ablation-zone size to manufacturer predictions and assessment of new antenna design

**DOI:** 10.1016/j.redii.2025.100061

**Published:** 2025-05-27

**Authors:** Théo Bonnefoy, Georges Tarris, Kévin Guillen, Olivia Poupardin, Olivier Chevallier, Ludwig Serge Aho Glele, Jean-Michel Correas, Romaric Loffroy

**Affiliations:** a Department of Vascular and Interventional Radiology, Image-Guided Therapy Center, centre hospitalier universitaire François-Mitterrand, 14, rue Paul-Gaffarel, BP 77908, 21000 Dijon, France; b Department of Pathology, centre hospitalier universitaire François-Mitterrand, 14, rue Paul-Gaffarel, BP, 77908, 21000 Dijon, France; c ICMUB Laboratory, CNRS UMR 6302, université de Bourgogne, 9, avenue Alain-Savary, 21000 Dijon, France; d Biossan Advancing Biomedical and Experimental Research, pôle agricole Auxois Sud, 21320 Créancey, France; e Department of Epidemiology, Statistics and Clinical Research, centre hospitalier universitaire François-Mitterrand, 14, rue Paul-Gaffarel, BP 77908, 21000 Dijon, France; f Department of Adult Radiology, hôpital Necker-Enfants-malades, 149, rue de Sèvres, 75015 Paris, France

**Keywords:** Microwave ablation, Percutaneous, Interventional radiology, In vivo animal model

## Abstract

**Aim:**

Percutaneous microwave ablation is an effective and minimally invasive treatment for small tumors. To achieve local disease control, the entire tumor and a surrounding safety margin must be destroyed. Power and application time are chosen based on manufacturer-provided data, usually obtained from ex vivo animal models. However, ex vivo tissues differ from in vivo condition due to compositional changes and absence of heat dissipation by blood flow. This study aimed to compare in vivo ablation zone sizes in swine with those predicted by the device manufacturer.

**Methods:**

Five pigs underwent 40 microwave ablation procedures using various power-time-organ combinations; 18 hepatic and 20 renal zones were evaluable. All procedures were performed with devices from a single manufacturer (ECO Microwave System Co, Nanjing, China). After euthanasia, the ablation zones were excised and sliced. For each ablation, the slice showing the largest dimensions was selected to measure x and y diameters and used to compute the ablated surface area.

**Results:**

For seven of eight power-time-organ combinations, significant differences were found between predicted and measured surface areas (*p* < 0.05), with deviations ranging from –45 % to +54 %. The overall mean absolute differences between measured and predicted ablation sizes in the x and y dimensions and the ablation surface area were 7.6 ± 4.6 mm (28 % ± 19 %), 5.8 ± 4.3 mm (18 % ± 13 %) and 273 ± 210 mm² (39 % ± 34 %), respectively.

**Conclusion:**

Manufacturer-provided predictive data for microwave ablation zone size may lack reliability. Intraoperative and postoperative monitoring of ablation zone size is crucial to ensure complete tumor destruction with adequate margins.

## 1 Introduction

Tumor thermal ablation is now an integral part of cancer management. Its minimally invasive nature offers advantages over conventional surgery, such as decreased morbidity and shorter hospital stays, while providing comparable outcomes for small tumors [1]. Thermal ablation is primarily used for unresectable hepatocellular carcinoma and liver metastases but is also an option for small primary kidney tumors and is increasingly applied to lung and bone tumors [[2], [3], [4], [5], [6], [7], [8], [9], [10]].

Both hepatocellular and renal cell carcinomas are common malignancies associated with substantial mortality [11,12]. The early detection and treatment of these tumors therefore produces major public health benefits. More specifically, hepatocellular carcinoma is often diagnosed based on imaging alone using the Barcelona criteria and treated without histological confirmation [13]. Moreover, nearly 50 % of renal carcinomas are asymptomatic but 90 % can be detected by CT [14].

Of the three main thermal ablation techniques, microwave and radiofrequency apply heat, whereas cryoablation causes ice formation via the delivery of a refrigerant gas. The more recent, non-thermal method known as irreversible electroporation uses high-voltage, pulsed, electrical fields to create nanopores in the cell membrane [15].

Microwave ablation involves the application of an electromagnetic field that oscillates polar molecules, such as water, causing frictional heating. Local heat production occurs in a more controlled manner with microwave than with radiofrequency ablation, particularly in water-rich and high-impedance tissues [1].

The main limitation of radiofrequency ablation is its susceptibility to the heat-sink effect, in which blood flow within nearby vessels dissipates the heat, reducing the ablation zone. Microwave ablation is less affected and can produce larger ablation volumes, especially near vessels [16]. Microwave ablation has the advantage of requiring a single energy-delivering antenna, although multiantenna techniques also exist, notably compared to cryoablation and irreversible electroporation. Microwave ablation does not induce systemic immune response syndrome, which is a well-documented complication of radiofrequency ablation used to treat large lesions. Furthermore, procedure time is significantly shorter with microwave ablation [17,18].

Complete tumor destruction with sufficient (5–10 mm) margins is crucial to prevent local recurrence. The size of the ablation zone is therefore a key consideration. Manufacturers of ablation devices provide charts that predict ablation-zone size according to the power applied and to the application time. However, most manufacturers obtain their predictive data from ex vivo animal samples, ECO® Microwave System Co used non-perfused organs from pigs at room temperature to establish those charts. Whether these data reflect in vivo ablation-zone size in humans is unclear.

In the interests of easier projection of ablation zones and better control of the local environment, manufacturers are increasingly tending to obtain ablation volumes as spherical as possible, microwave ablation typically produces ellipsoid ablation due to a wider propagation of energy along the axe of the antenna.

The primary objective of this study was to determine whether ablation-zone sizes after in vivo hepatic and renal microwave ablation in pigs differed significantly from the sizes predicted by the device manufacturer. A secondary objective was to determine ablation zone sizes obtained using a new type of microwave antenna (ECO-200 G, ECO Microwave System Co, Nanjing, China) claimed by its manufacturer to produce ablation surface areas of up to 48 × 50 mm.

## 2 Material and methods

### 2.1 Animals

The study was conducted in compliance with the European directive on animal experimentation (Directive EU/2010/63). We studied five, female, large white pigs (Sus scrofa domesticus) purchased from GAEC Boccard, Auxan, France. For each, age was between 4 and 6 months (Table 1). The livers and kidneys of pigs and humans share many similarities in terms of morphology, size, and vascularization. Moreover, coagulation is closely similar in the two species.

**Table 1:** In vivo percutaneous microwave ablation with ECO system in swine kidney and liver: comparison of ablation-zone size to manufacturer predictions: main characteristics of the five study animals.

Characteristics	Mean ± SD	Range
Weight (kg) (5 animals)	73 ± 2	70–76
Operating time (h) (40 procedures)	4 ± 1.1	3–6
Systolic blood pressure (mmHg)		
Pig 1	104 ± 10	83–124
Pig 2	121 ± 15	98–148
Pig 3	118 ± 11	93–139
Pig 4	111 ± 13	87–134
Pig 5	110 ± 11	93–125

SD: standard deviation.

Throughout the procedure, systolic blood pressure, heart rate, and peripheral oxygen saturation were monitored closely to ensure optimal hemodynamic stability. All five animals exhibited satisfactory perfusion of the target organs defined by a constant cardiac activity and hemodynamic stability. Target-organ perfusion is a major difference between in vivo and ex vivo microwave ablation and may alter the size of the ablation zone.

### 2.2 Procedures

After intramuscular administration of 0.4 mg/kg of the sedative neuroleptic azaperone (Stresnil®, Elanco, IN), each animal was placed in the prone position. Anesthesia was induced by an intramuscular injection of 5 mg/kg tiletamine hydrochloride/zolazepam hydrochloride (Zoletil® 100, Virbac AH, Carros, France). The animal was intubated and anesthesia maintained by isoflurane inhalation (Isoflu-Vet®, DECHRA Veterinary Products SAS, Montigny-le-Bretonneux, France).

Saline was given through a catheter placed in an ear vein. This catheter was used to administer 3 mg/kg ketoprofen (Ketofen® 10 %, Ceva, Marseille, France) to limit lung macrophage demargination and 0.1 mg/kg butorphanol (Butador®, Boehringer Ingelheim, Ingelheim, Germany) to prevent pain. An arterial catheter was placed to allow continuous blood pressure monitoring. Finally, a urinary catheter was inserted.

**Fig. 1:**
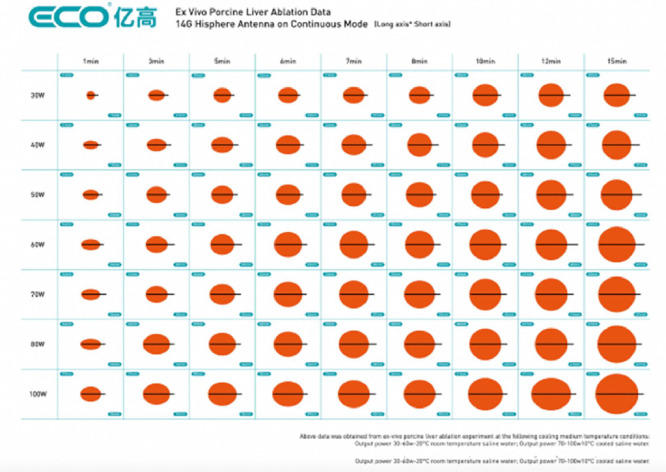
In vivo percutaneous microwave ablation with ECO system in swine kidney and liver: comparison of ablation-zone size to manufacturer predictions. Example: Hisphere 14 G needle for liver ablation^a^.

^a^The data are from ex vivo porcine liver ablation with the following cooling temperatures: output power, 30–60 W and saline at room temperature (20 °C); output power, 70–100 W and cooled saline; output power, 30–60 W and saline at room temperature (20 °C); and output power, 70–100 W and cooled saline (10 °C).

The microwave generator (ECO-200 G) and antennas were from a single manufacturer (ECO Microwave System Co, Nanjing, China). The generator was used at an operating frequency of 2.45 GHz, in continuous and pulsed modes with standard and spherical antennas, respectively. Cooling system using NaCl solution at room temperature (20 °C) with safety shutdown if solution temperature exceeds 35 °C. Each procedure was performed using a single antenna, which was inserted by an experienced interventional radiologist (RL or JMC) under ultrasound guidance (Aplio i800, Canon Europe, Uxbridge, Middlesex, UK). At the liver, all ablations were with the spherical ECO-100CL8C HiSphere antenna. In the kidney, either the recently introduced, new spherical ECO-100CL5C HiSphere antenna or a standard antenna was used. Power and time were selected based on the ablation-zone size predicted by the manufacturer for the type of antenna used (Table 2**,** Fig. 1). The ablation procedures were performed in random order to limit bias, before each ablation an organ and an ablation parameter were randomly selected within the limits of four hepatics ablations and four kidney ablations for each pig, total number of ablations for each parameter was not adjusted between each pig.

**Table 2:** In vivo percutaneous microwave ablation with ECO system in swine kidney and liver: comparison of ablation-zone size to manufacturer predictions: microwave ablation parameters by needle type and organ.

Antenna	Organ	Designation^a^ (number of microwave ablations)	Power (W)	Time (min)	Surface area predicted (mm × mm)
ECO-100 CL9C HiSphere 14G	Liver	A (7) B (5) C (6)	30 40 80	5 8 10	19 × 22 30 × 31 40 × 40
ECO-100CL5C HiSPhere 16G	Kidney	D (2) E (2) F (4)	30 45 60	8 8 12	21 × 22 NA 36 × 37
ECO-100CL5C Standard 16G	Kidney	G (5) H (4) I (3)	30 40 50	5 8 8	23 × 36 33 × 47 35 × 50

NA: not available.

a Each power-time-organ combination is designated by a letter.

### 2.3 Ablation-zone size measurement

**Fig. 2:**
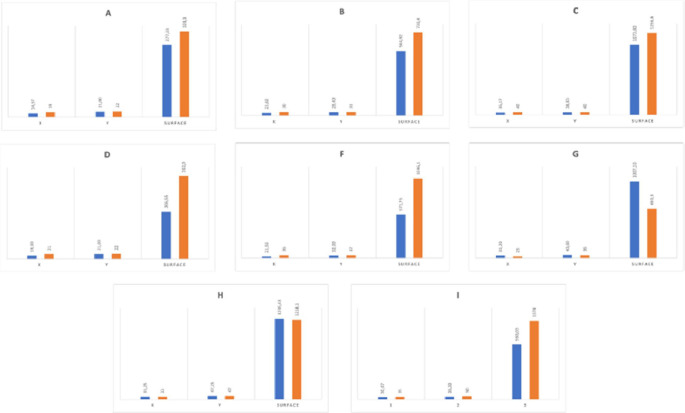
In vivo percutaneous microwave ablation with ECO system in swine kidney and liver: comparison of ablation-zone size to manufacturer predictions: mean measured values of x and y in millimeters and surface area in square millimiters are shown in blue and corresponding predicted values are in orange. The E parameter combination is not shown, due to the absence of predictive data.

**Fig. 3:**
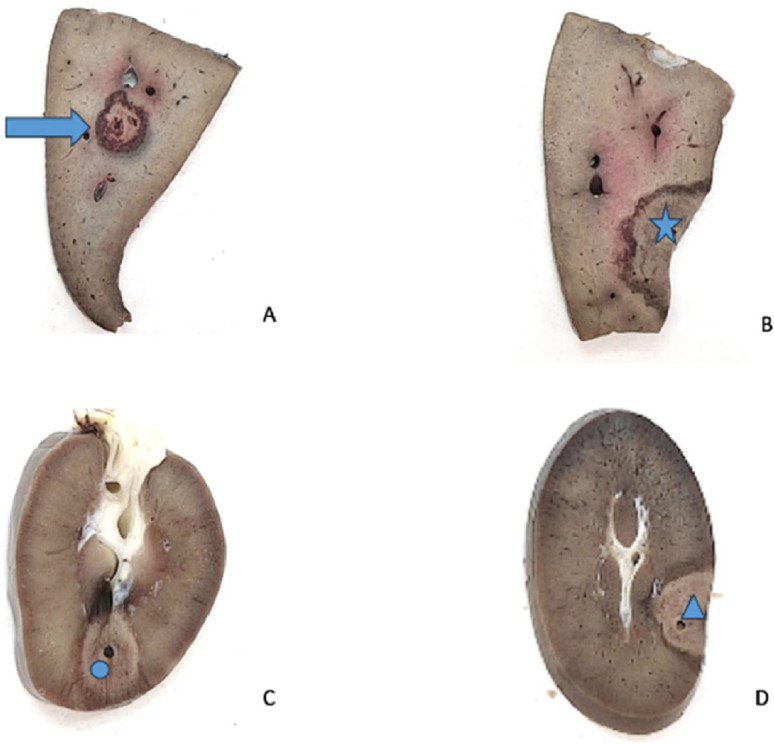
In vivo percutaneous microwave ablation with ECO system in swine kidney and liver: comparison of ablation-zone size to manufacturer predictions: macroscopic appearance of ablation zones, four examples.

**Fig. 4:**
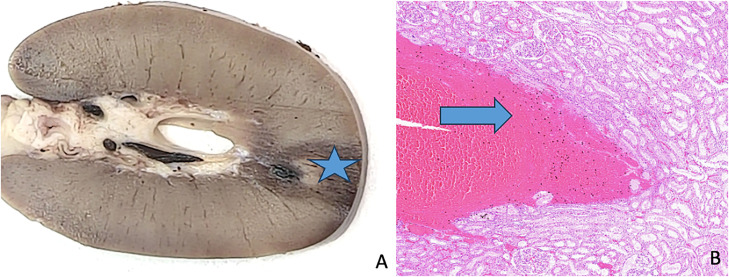
In vivo percutaneous microwave ablation with ECO system in swine kidney and liver: comparison of ablation-zone size to manufacturer predictions: example of a renal ablation zone.

The animals were euthanized by intra-venous administration of sodium pentobarbital, 0.1 mL/kg (Euthasol-Vet®, DECHRA Veterinary Products SAS, Montigny-le-Bretonneux, France) immediately after the last MWA procedure and dissected, procedure times from anesthesia to dissection range from 03 h 50 (Pig 5) to 06 h 35 (Pig 1), all dissection were performed under the same among of time, within one hour of the euthanasia. The ablation zones were identified macroscopically by targeting the characteristic necrosis zones in cocarde shape and, if needed, by ex vivo ultrasound and hand palpation of hard nodule. Each zone was excised and fixed in formalin (Fig. 2). Fig. 3, Fig. 4 show examples of ablation zones.

A. Complete hepatic tissue ablation (arrow), B. Incomplete hepatic tissue ablation (star), C. Complete renal tissue ablation (circle), D. Incomplete renal tissue ablation (triangle)

A. Macroscopic section: the star indicates the ablation zone, B. Microscopic features: the arrow indicate the necrosis

For the macroscopic evaluation, each sample was cut by a qualified pathologist to ensure accurate and reproducible measurement of each ablation zone. The cuts were perpendicular to the major axis of the interest aera to minimize measurement bias, especially if ablation zone was ellipsoid which can lead to an artificial increase in surface measurement if the cut is not perpendicular to the major axis. Image J software was then used to measure the length and width (x and y) of ablation zone contained within each slice [19]. Care was taken to include the slice through the largest macroscopic ablation zone. The surface area of the largest ablation zone was computed using the mathematical formula appropriate for the shape of the zone, i.e., circular or elliptical.

The microscopic study was carried out on nine, randomly selected ablation zones. The percentages of each zone with necrosis and thermocoagulation were determined.

### 2.4 Statistical analysis

Normal distribution of each variable (x, y, area) has been demonstrated by a Kolmogorov-Smirnov test. Continuous quantitative variables (x, y, surface area) were described as mean ± SD and compared by applying the linear regression *t*-test regardless of the ablations’ parameters used, statistical studies cannot be applied independently to each parameter due to the small number of data. The statistical analyses were done using STATA version 15.1 (StataCorp, College Station, TX).

## 3 Results

### 3.1 Animals and procedures

Table 1 shows the body weight, procedure time, and systolic blood pressure in the five animals. Body weight and systolic blood pressure during the procedures showed only limited variability across animals.

Forty microwave ablation procedures were performed, four in the liver and two in each kidney of each pig. Table 2 reports the combinations of power and time for the liver and kidney and for each antenna design. Two liver ablation zones were excluded because they were located by mistakes in the spleen; the procedures in these two zones were done using the B and C parameter combinations in Table 2.

For the procedures done with the E parameter combination, no predictive data were available from the manufacturer, as the antenna had been only recently introduced. Consequently, the ablation-zone sizes for the procedures done with E were not included in the statistical analysis.

Due to the randomization, the number of procedures differed across parameter combinations (Table 2).

### 3.2 Safety

Only one complication was reported in subject number five, a medium-abundance hemoperitoneum for which the primary hemorrhagic site was not determined, possibly related to the abdominal wall dissection as it was not detected during the per-procedure ultrasonography and no hemodynamic instability was reported prior to euthanasia.

Of the 38 ablation zones, 21 were located near the hepatic or renal capsule, no evidence of tissue destruction was found in the structures located outside the liver or kidney near the ablation target. Euthanasia was necessary due to the large areas of ablation within the target organs and the need to remove large volumes of tissue that contained all the ablation zones, the functional integrity of the organs could not be preserved.

### 3.3 Macroscopic findings

By univariate analysis, the x and y dimensions and the ablation surface areas differed significantly from those predicted by the manufacturer (*p* = 0.0001 for all comparisons) (Table 3 and Fig. 2).

**Table 3:** In vivo percutaneous microwave ablation with ECO system in swine kidney and liver: comparison of ablation-zone size to manufacturer predictions: distances on the x and y axes and of surface areas.

Parameter designations	x predicted (mm)	y predicted (mm)	Surface area predicted (mm²)	x measured mean ± SD (mm)	y measured mean ± SD (mm)	Surface area measured mean ± SD (mm²)
A	19	22	328	14 ± 9	21 ± 7	277 ± 297
B	30	31	730	23 ± 5	29 ± 8	564 ± 236
C	40	40	1256	35 ± 1	39 ± 4	1074 ± 128
D	21	22	363	13 ± 3	21 ± 7	206 ± 26
E	NA	NA	NA	19 ± 1	22 ± 1	327 ± 3
F	36	37	1046	21 ± 2	32 ± 7	571 ± 20
G	23	36	650	31 ± 5	40 ± 7	1007 ± 275
H	33	47	1218	33 ± 7	47 ± 4	1235 ± 312
I	35	50	1374	31 ± 8	38 ± 11	960 ± 448

SD: standard deviation; NA: not available.

Table 4 reports the differences, as percentages, between the measured and predicted ablation-zone surface areas. Substantial differences were found, except with parameter combination H . With one parameter combination (G), the measured surface area was much larger than predicted. For the remaining six parameter combinations, the measured surface areas were smaller than predicted, and the differences were at times considerable, i.e., for D and F, the two kidney procedures with the new 16 G HiSphere antenna. Using a standard antenna instead (G, H, and I) had widely variable effects.

**Table 4:** In vivo percutaneous microwave ablation with ECO system in swine kidney and liver: comparison of ablation-zone size to manufacturer predictions: difference between predicted and measured surface areas^a^.

Designation^b^	Power (W)	Time (min)	Predicted SA (mm²)	Measured SA mean ± SD (mm²)	Difference mean ± SD ( %)
A	30	5	328	277 ± 297	- 15 ± 85
B	40	8	730	564 ± 236	- 22 ± 32
C	80	10	1256	1073 ± 128	- 14 ± 10
D	30	8	363	206 ± 26	–43 ± 7
F	60	12	1046	571 ± 20	–45 ± 2
G	30	5	650	1007 ± 275	+ 54 ± 42
H	40	8	1218	1235 ± 312	+ 2 ± 26
I	50	8	1374	960 ± 448	–30 ± 33

SA: surface area; NA: not available.

a The E parameter combination is not shown, as no predictive data were available.

b Each power-time-organ combination is designated by a letter.

Of the 36 ablations included in the statistical data, 21 were performed in the juxtacapsular zone, which led to an underestimate of their surface area because at least one of the two x or y axes was not fully included in the measurement, this concerned 29 % of ablations for parameter A, 40 % for B, 17 % for C, 100 % for D, 75 % for F, 100 % for G, 100 % for H and 70 % for I, i.e., 28 % of all liver ablations and 89 % of all kidney ablations. The overall mean absolute differences between measured and predicted ablation sizes in the x and y dimensions, as well as the ablation surface area, were 7.6 ± 4.6 mm (28 % ± 19 %), 5.8 ± 4.3 mm (18 % ± 13 %) and 273 ± 210 mm² (39 % ± 34 %), respectively. For liver ablations, the mean differences between measured and predicted ablation sizes in the x and y dimensions, as well as the ablation surface area, were respectively 5.3 ± 7.0 mm (22 % ± 34 %), 1.3 ± 7.4 mm (5 % ± 30 %) and 99 ± 257 mm² (19 % ± 66 %), respectively. The corresponding mean absolute differences were 7.3 ± 4.6 mm (33 % ± 23 %), 6.1 ± 4.0 mm (24 % ± 16 %) and 208 ± 172 mm² (50 % ± 45 %). For kidney ablations, the mean differences were respectively 3.1 ± 8.6 mm (7 % ± 30 %) in the x dimension, 1.3 ± 7.2 mm (3 % ± 18 %) in the y dimension, and 112 ± 366 mm² (7 % ± 43 %) in surface area. The mean absolute differences were 7.8 ± 4.7 mm (25 % ± 17 %), 5.6 ± 4.5 mm (15 % ± 10 %) and 305 ± 224 mm² (34 % ± 26 %), respectively.

The two ablations done with the E parameter combination, for which predictive data were unavailable, produced similar ablated surface areas of 330 mm² and 325 mm², respectively.

Of the 36 ablations carried out, accepting a macroscopic difference between the x and y axes of ± 1 mm, we obtained six spherical ablation surfaces and 30 ellipsoids.

### 3.4 Microscopic findings

Only nine ablation zones were examined histologically examination. The results varied widely, with the proportion of ablation zone containing necrosis and thermocoagulation ranging from 8 % to 100 % and from 0 % to 100 %, respectively.

## 4 Discussion

Our in vivo study of 18 liver and 20 kidney microwave ablation procedures in five pigs with ex vivo determination of ablation-zone size by pathological examination used a variety of power-time combinations and three antenna designs. Ablation-zone size was often substantially different from the size predicted by the manufacturer based on procedures on ex vivo animal organs. These major differences are of concern. In most instances, the ablated zone was smaller than predicted, although in one case it was 54 % larger. None of the animals experienced death or major complications.

Another study used a similar design to ours, comparing ablation-zone sizes predicted from ex vivo bovine hepatic microwave ablation to the sizes obtained during 18 in vivo renal procedures in three pigs [20]. When a single antenna was used, the difference in greatest ablation zone diameter between the in vivo procedures and the predictions ranged from +40 % to –47.05 % The mean difference was −8.6 % ± 30.1 %, with the in vivo ablation zones being smaller. Similarly, in 20 patients who underwent 25 procedures to treat liver tumors, the ablation zones assessed immediately postablation by CT were significantly smaller than the ex vivo reference values provided by the manufacturers [21].

In our study, the differences between measured and predicted values were greatest with the D and F parameter combinations, which consisted in 30 W-8 min and 60 W-12 min, respectively, at the kidney, with a spherical antenna designed to produce spherical rather than elliptical ablation volumes which is theoretically more acceptable due to its ease of modelling in order to guarantee the best processing conditions. The surface areas were 43 % and 45 % smaller, respectively, than predicted. One possible explanation is an interaction between the ultrasound waves and the coating present on these antennas. Another hypothesis is that the pulsed emission that creates a spherical ablation zone may deliver less energy or be more quickly inhibited by the blood flow to the target, particularly in highly vascular organs such as the kidney, compared to the emissions that create ellipsoids.

The reason why one specific parameter combination G (standard antenna, 30 W, 5 min, kidney) resulted in larger-than-expected ablation zones remains unclear. One possible explanation is that the ablation zone predicted for this parameter set was underestimated by the manufacturer. It is likely that multiple factors contributed to these results. Given that the kidney is a highly perfused organ, it is also possible that hemodynamic variations contributed to larger ablation zone. As with combinations H and I, a standard antenna was used with combination G, and ablations were performed in the same three animals (pigs 3, 4, and 5). Compared to these two other groups, combination G involved the lowest power (30 W) and the shortest application time (5 min). Surprisingly, however, this combination resulted in even larger ablation zones than combination I, which used higher power (50 W) and a longer application duration (8 min). For combinations G and H, 100 % of the ablations were performed in the juxtacapsular zone, compared to 70 % for combination I. Therefore, the larger average ablation zones observed with combination G may be partly explained by a more peripheral location, whereas combination I included more centrally located ablations, closer to larger vessels, which are likely to have induced a stronger heat sink effect. Furthermore, given the smaller expected ablation size with combination G, an unintentional slightly more peripheral positioning of the antenna cannot be ruled out.

Simulation platforms are being evaluated for microwave ablation procedures. A simulation model based on CT images segmented by a clinician and taking into account the density, dielectric properties, thermal conductivity, and the heat capacity of the healthy and tumor tissues was created for two liver tumors in human patients [22]. A computational model of microwave ablation based on finite element modeling has also been reported [23]. Such models could be used to determine the optimal power and time parameters for microwave ablation. A 2022 review supports the usefulness of these numerical approaches [24].

In the IAMCOMPLETE study, coregistration of pre- and postablation CT images was performed using a rigid registration algorithm to quantify ablation margins following hepatocellular carcinoma radiofrequency or microwave ablation. This approach was deemed feasible in 16 out of 20 patients and for 26 out of 31 tumors [29]. Local tumor progression occurred in four patients, each involving one of the ablated tumors. This included one case where treatment was unsuccessful due to early termination from bleeding, one tumor that was invisible on both ultrasound and preablation CT, and two tumors with a minimal ablation margin of –4.00 mm. Interestingly, none of the patients with a confirmed minimal ablation margin greater than 0 mm developed local tumor progression. This study highlights the importance of immediate postprocedural assessment of the ablation zone and margins on CT imaging to anticipate treatment efficacy and guide early management decisions.

With recent technological advancements and the development of robot-assisted techniques, robot-guided needle placement may improve the outcomes of tumor ablation procedures. In a recent study, the use of robotic systems for the ablation of 48 abdominal tumors was shown to be feasible, safe, accurate, and effective [30].

One limitation of our study is the limited sample size. The three Rs principle for animal experiments includes restriction of the number of animals used. Second, the livers and kidneys were healthy and apparently free of abnormalities. Energy propagation is affected by tissue composition, particularly in water-rich tissues, and by local blood flow. Tumor tissue is usually highly vascularized, a feature that might create different ablation-zone sizes from those recorded in our study [25,26]. Third, we used a macroscopic technique to measure ablation-zone size. Other methods such as shear-wave imaging or microwave tomography may be more accurate but were not available to us [27,28]. Some ablation zones were located adjacent to the hepatic or renal capsule, that is, not entirely surrounded by parenchyma. The size of these zones may have been underestimated by our technique. We measured surface area and not volume. Moreover, we did not assess the intraobserver and interobserver reproducibility of our ablation-zone measurement method. Fourth, microscopic studies were done on only nine of the 38 ablation zones. The proportions of tissue with necrosis and thermocoagulation varied widely. The tissue fixation procedure may have affected the measurements. Also, ablated tissue is difficult to distinguish microscopically from the thermocoagulation tail. However, the microscopic appearance of the ablated tissue was not the focus of our study. Fifth, we assessed prediction data from a single manufacturer. Sixth, as the animals were euthanized after the final ablation, major complications and ablation-related deaths may have been underestimated. Finally, we studied a single animal species, for which correlations of ablation-zone sizes with those in humans have not been determined. Sixth, for the C parameters ablations the constructor recommends a cooled saline at 10 °C where we used a saline at room temperature (20 °C).

## 5 Conclusion

Although microwave ablation is now an established method for treating tumors at several sites, the ablation zone sizes predicted by manufacturers remain unreliable, with effective zones being frequently falling short of the predicted dimensions. Our study highlights the crucial importance of accurate ablation-zone size monitoring during the procedure to achieve destruction of the entire tumor and of a sufficient safety margin. Post-treatment evaluation is also important to determine whether a further procedure is needed. Finally, we provide preliminary information on the ECO-100CL5C HiSphere 16 G antenna, which requires further evaluation.

## Funding

This research received external funding from ECO Microwave System Co, Nanjing, China.

## Informed consent statement

Not applicable for this animal study.

Data Availability Statement: The data presented in this study are available on request from the corresponding authors. The data are not publicly available due to identity reason.

## Institutional review board statement

The animal study protocol was approved by the Comité d’éthique de l’expérimentation animale grand campus Dijon (protocol code APAFIS #37,503–2022,051,918,223,550 v1).

## Author contributions

Conceptualization: J.M.C. and R.L; Data curation: K.G.; Formal analysis: L.S.A.G; Funding acquisition: R.L.; Investigation: R.L.; Methodology: T.B., G.T., K.G., J.M.C. and R.L.; Project administration: O.P.; Resources: O.P.; Software: O.P.; Supervision: R.L.; Validation: O.C., J.M.C. and R.L.; Visualization: R.L.; Writing - original draft: T.B., KG and R.L.; Writing - review & editing: T.B., G.T., K.G., O.C., L.S.A.G., J.M.C. and R.L.

All authors have read and agreed to the published version of the manuscript.

## Declaration of competing interest

The authors declare the following financial interests/personal relationships which may be considered as potential competing interests: RL reports financial support was provided by ECO Microwave System Co, Nanjing, China. If there are other authors, they declare that they have no known competing financial interests or personal relationships that could have appeared to influence the work reported in this paper.
